# Molecular investigation and genetic characterization of feline leukemia virus (FeLV) in cats referred to a veterinary teaching hospital in Northern Italy

**DOI:** 10.1007/s11259-024-10380-6

**Published:** 2024-04-22

**Authors:** Laura Gallina, Veronica Facile, Nicola Roda, Maria Chiara Sabetti, Alessia Terrusi, Lorenza Urbani, Martina Magliocca, Kateryna Vasylyeva, Francesco Dondi, Andrea Balboni, Mara Battilani

**Affiliations:** 1 Department of Veterinary Medical Sciences, Alma Mater Studiorum-University of Bologna, Via Tolara di Sopra 50, Ozzano Emilia, Bologna 40064 Italy; 2 Department of Veterinary Sciences, University of Parma, Strada del Taglio 10, Parma, 43126 Italy

**Keywords:** FeLV, Subtypes, Cat, Retrovirus, Point-of-care test, Provirus, Real-time PCR

## Abstract

Feline leukemia virus (FeLV) is responsible for feline leukemia syndrome in domestic cats. The prevention and control of disease caused by FeLV are primarily based on vaccination and identification and isolation of infected subjects. Antigen diagnostic methods, which are the most widely used in clinical practices, can be associated to molecular tests to characterize the FeLV detected. In this study, a quantitative SYBR Green Real-Time PCR (qPCR) assay was used to detect FeLV proviral DNA in blood samples from antigen positive cats referred to a veterinary teaching hospital in Northern Italy in 2018–2021. To genetically characterize the identified viruses, a portion of the viral envelope (*env*) gene was amplified using six different end-point PCRs and sequenced. Twenty-two of 26 (84.6%) cats included in the study tested positive by qPCR assay. This suggests a high performance of the qPCR adopted but further studies are required to investigate the cause of discordant results between the antigen test and qPCR in four cats. From *env* gene analysis, 15/22 qPCR-positive cats were infected by FeLV subtype A and 5/15 shown coinfection with subtype B.

**Supplementary Information:**

The online version contains supplementary material available at 10.1007/s11259-024-10380-6.

## Introduction

Feline leukemia virus (FeLV) is an enveloped RNA virus belonging to the genus *Gammaretrovirus* occurring worldwide in domestic and small wild cats. In Italy, a recent study estimated a prevalence of FeLV infection of 5.7% (Studer et al. 2019). FeLV genome can integrate into the host cell genome as provirus and may affects the cellular replication, leading to neoplastic transformation and immunosuppression. Endogenous FeLV (enFeLV), in cats’ genome, have been correlated with resistance against exogenous virus (exFeLV) infections and play a crucial role in arising FeLV recombinant subtypes (Stewart et al. 1986; Roy-Burman 1996). The longest known FeLV subtypes are FeLV-A, FeLV-B, and FeLV-C. More recently, FeLV subtypes D, T and TG35 have been identified (Chiu et al. 2018). FeLV-A is the most widespread since it is the only subtype transmissible from cat to cat and it is present in every infected cat.

The clinical outcome in cats is determined by a combination of viral and host factors such as the FeLV subtypes involved, the age of the cat at the time of infection and its immune response. According to this, FeLV infection has four possible outcomes: progressive, regressive, abortive and rarely focal or atypical infection (Little et al. 2020).

The understanding of the infectious status of cats is important before vaccination or to identify and isolate infected subjects within naive multi-cat environments. Point-of-care (PoC) tests based on the detection of p27 antigen of FeLV are widely used to identify infected cats. PoC tests are very performant to detect cats with progressive and focal (atypical) infection, but they show limitations in case of the regressive infections for which the use of molecular methods like PCR assay to detect proviral DNA is recommended (Hofmann-Lehmann and Hartmann 2020). Moreover, the diagnosis of FeLV is very difficult because of the complex pathophysiology and evolution that the infection can have, for these reasons it is not possible to use a single test to determine the status of FeLV infection (Beall et al. 2021; Giselbrecht et al. 2023).

The aims of this study were to detect FeLV provirus in whole blood samples of antigen-positive cats from northern Italy and to genetically characterize the identified viruses.

## Materials and methods

Cats tested positive for the presence of FeLV specific antigens were included in the study. All samples were collected between January 2018 and September 2021 at the Veterinary University Hospital (VUH) of the University of Bologna. Plasma, serum, or whole blood samples were tested for FeLV p27 antigen and Feline immunodeficiency virus (FIV) antibodies using a commercial PoC enzyme linked immunosorbent assay (ELISA) based test (SNAP FIV/FeLV Combo Plus test, IDEXX, USA). PoC tests were carried out within one hour from the sampling and the samples were stored at − 20 °C after examination.

Signalment data, clinical signs and clinicopathological findings of enrolled cats were retrieved from medical records. The enrolled cats were grouped as: (i) “symptomatic cats” with clinical signs or clinicopathological abnormalities referable to FeLV infection (SC group) and (ii) “asymptomatic cats” without clinical signs or clinicopathological abnormalities referable to FeLV infection (AC group).

Genomic DNA extraction from K_3_EDTA blood samples was carried out starting from 200 µl of sample and the DNA was eluited in 100 µl of elution buffer, using a commercially available kit (NucleoSpin Tissue Kit, Macherey-Nagel, Germany).

The presence of FeLV DNA was screened by using a quantitative SYBR Green Real-Time PCR (qPCR) assay (Tandon et al. 2005) (Online Resource 1). The reaction was performed using the PowerUp SYBR Green master mix (Thermo Fisher Scientific, USA) following manufacturer’s instructions. FeLV DNA copies number determination was carried out by absolute quantification using tenfold dilutions of a DNA standard plasmid containing 468 nucleotides sequence from the U3 region (Online Resource 1), cloned into the TOPO TA Cloning vector (Invitrogen, USA). In each reaction, seven tenfold dilutions of the DNA standard plasmid and samples were amplified in duplicates together with a no template control.

Six previously published end-point PCR assays were used to amplify fragments of different length of the FeLV *env* gene from the qPCR-positive cats. End-point PCRs 1 to 4 were reported to target FeLV A, B and C while PCRs 5 and 6 were reported to be specific only for FeLV B (Erbeck et al. 2021; Watanabe et al. 2013). Reactions were carried out with the proofreading Phusion Hot Start II High-Fidelity DNA Polymerase (Thermo Fisher Scientific, USA), according to the manufacturer’s instructions.

The *env* gene PCR products were sequenced by Sanger method using the forward and reverse primers adopted for the amplification plus primers designed for sequencing (Online Resource 1). The sequences were assembled with CodonCode Aligner software, aligned with 34 reference sequences retrieved from GenBank database (Online Resource 2) and translated into amino acid sequences using the ClustalW method implemented in the BioEdit 7.0.5 software. Nucleotide and amino acid similarity of obtained sequences with reference sequences and potential recombination breakpoints were evaluated to determine which FeLV subtype the identified viruses belonged to. The assembled nucleotide sequences were analyzed using the BLAST web interface (https://blast.ncbi.nlm.nih.gov/Blast.cgi). Phylogeny was carried out on *env* gene nucleotide sequence alignment using MEGA 11 version 11.0.10 (Tamura et al. 2021).

Data were evaluated using standard descriptive statistics and reported as median and range. Categorical data such as year of sampling, sex, breed and antibodies test results were analyzed using the Fisher’s exact P-value test or Pearson’s Chi-squared test, while continuous data (age) were analyzed by the Mann-Whitney U test. Statistical significance was set at *P* < 0.05. Statistical analysis was carried out using the MedCalc Statistical Software version 16.8.4 (MedCalc Software bvba).

## Results

Twenty-six FeLV antigen-positive cats were included in the study. Signalment data, clinical signs, clinicopathological findings and FIV antibodies PoC test results are reported in Tables 1 and 2. Eighteen of 26 (69.2%) cats were included in the SC group and 8/26 (30.8%) were included in the AC group. No statistical association was found regarding the presence of clinical signs or clinicopathological abnormalities associated with FeLV infection and signalment data.

**Table 1:** Descriptive statistics and grouping of FeLV antigen-positive cats included in this study

Variables		Total	SC	AC	P value
Number of cats		26	19 (673.1%)	7 (26.9%)	
Year of sampling					
	2018	5 (19.2%)	5 (26.3%)	0 (0%)	0.2966
	2019	9 (34.6%)	5 (26.3%)	4 (57.1%)	
	2020	6 (23.1%)	4 (21.1%)	2 (28.6%)	
	2021	6 (23.1%)	5 (26.3%)	1 (14.3%)	
Sex					
	Male	15 (57.7%)	8 (42.1%)	7 (100%)	0.0276
	Female	11 (42.3%)	11 (57.9%)	0 (0%)	
Age ^a^		6y1m [6 m-15y11m]	5y5m [10 m-14y7m]	4y7m [6 m-15y11m]	0.8322
Breed					
	DSH	26 (100%)	19 (100%)	7 (100%)	
	Purebred	0 (0%)	0 (0%)	0 (0%)	
FIV antibodies test					
	Positive	4 (15.4%)	1 (5.3%)	3 (42.9%)	0.0812
	Negative	22 (84.6%)	18 (94.7%)	4 (57.1%)	

The chi-squared test and the Mann-Whitney U test (age) were carried out on the symptomatic and asymptomatic cats. Data are reported as n (%). AC: asymptomatic cats group. DSH: domestic short-hair cat. m: months. SC: symptomatic cats group. y: years

^a^ Data are reported as median [range]

**Table 2:** Signalment data, clinical manifestations, year of sampling, result of molecular test and viral *env* gene sequences of PoC test positive cats included in the study

Cat	Year of sampling	Sex	Age	FIV	Group	Clinical manifestations referable to FeLV infection	Concomitant diseases	LTR qPCR result (Ct value)	Nucleotide sequence (*env* gene)	GenBank ID	Sequence length (nucleotides)
320	2018	M	14y	Neg	SC	Anorexia, wobble, cough, suspected renal lymphoma	Sialocele	Pos (19)	Subtype A	OR227248	1800
									Endogenous	OR227249	1272
321	2018	FS	4y 7 m	Neg	SC	Anaemic syndrome	Shock	Pos (21)	Subtype A	OR227250	1813
									Endogenous	OR227251	1298
322	2018	MC	10y 5 m	Neg	SC	Non-regenerative anaemia		Pos (21)	Subtype A	OR227252	1805
									Endogenous	OR227253	1295
327	2018	FS	2y 1 m	Neg	SC	Dyspnoea, depression, suspected lymphoma/thymoma, pleural effusion		Pos (18)	Endogenous	OR227254	1296
328	2018	M	14y 7 m	Neg	SC	Anorexia, cough, pulmonary mass		Pos (19)	Subtype A	OR227255	1850
									Subtype B	OR227256	1326
308	2019	MC	9y 2 m	Pos	SC	Anorexia, haemorrhagic stomatitis, rhinitis, anaemia		Pos (34)	Subtype A	OR227257	1858
									Endogenous	OR227258	1295
316	2019	FS	9y 10 m	Neg	SC	LGL (large granular lymphocyte) lymphoma		Pos (34)	Endogenous	OR227259	1308
319	2019	FS	9y 1 m	Neg	SC	Anorexia, anaemia, suspected leukaemia	Congestive heart failure	Pos (18)	NA	NA	
1128	2019	FS	8y 5 m	Neg	SC	CKD, inflammatory anaemia	Suspected hepatic lipidosis	Neg	NA	NA	
1199	2019	FS	3y	Neg	SC	IMHA, depression, atypical juvenile lymphadenitis	Cardiopathy, pleural effusion	Pos (35)	NA	NA	
1122	2020	FS	2y 8 m	Neg	SC	Hyperthermia, wobble, ataxia		Pos (18)	Subtype A	OR227269	1797
1123	2020	MC	6y 2 m	Neg	SC	Cough, pleural effusion, lymphadenopathy, suspected neoplasia	Trauma	Pos (19)	Subtype A	OR227270	1812
									Subtype B	OR227271	1206
1126	2020	MC	4y 6 m	Neg	SC	Hyperthermia, depression, PIMA		Neg	NA	NA	
1198	2020	MC	1y 5 m	Neg	SC	Hyperthermia, dysorexia, anaemia	Triaditis	Pos (21)	NA	NA	
324	2021	FS	2y	Neg	SC	Dysorexia, leucemic lymphoma		Pos (17)	Subtype A	OR227275	1680
									Subtype B	OR227276	1626
325	2021	M	7y 9 m	Neg	SC	Anorexia, anaemia, suspected round-cell neoplasia		Pos (37)	NA	NA	
732	2021	MC	10 m	Neg	SC	PIMA, depression, anorexia		Neg	NA	NA	
868	2021	FS	NA	Neg	SC	Non regenerative anaemia, depression		Neg	NA	NA	
1180	2021	M	1y 6 m	Neg	SC	PIMA	Cholangitis	Pos (21)	Subtype A	OR227280	1798
317	2019	MC	4y 8 m	Neg	AC		Brachial plexus pathology	Pos (19)	Subtype A	OR227260	1800
									Endogenous	OR227261	1293
323	2019	M	3y 7 m	Pos	AC		Investment trauma	Pos (22)	Subtype A	OR227262	1812
									Subtype B	OR227263	1292
1127	2019	M	15y 11 m	Pos	AC		Bite trauma, septic shock	Pos (24)	Subtype A	OR227264	1792
									Endogenous	OR227265	1282
1129	2019	M	6 m	Neg	AC		Investment trauma	Pos (20)	Subtype A	OR227267	1807
									Endogenous	OR227268	1293
1124	2020	M	1y 2 m	Neg	AC		Tarsal injury	Pos (15)	Subtype A	OR227272	1814
									Subtype B	OR227273	1294
1125	2020	MC	4y 7 m	Pos	AC			Pos (32)	NA	NA	
326	2021	M	11y 1 m	Neg	AC		Bite trauma	Pos (21)	Subtype A	OR227277	1801
									Endogenous	OR227278	1278

AC: asymptomatic cats group. F: female. FIV: result to Feline immunodeficiency virus antibodies PoC ELISA based test (SNAP FIV/FeLV Combo Plus test, IDEXX, USA). FS: sterilized. IMHA: immune-mediated haemolytic anaemia. M: male. MC: castrated male. m: months. NA: not available. Neg: negative. PIMA: precursor-targeted immune-mediated anaemia. PoC: point-of-care. Pos: positive. SC: symptomatic cats group. y: years

Twenty-two of 26 (84.6%) cats tested positive by qPCR (Table 2). The quantity of FeLV DNA varied between 2.8 and 3 × 10^6^ copies of target amplicon/µL of extract, that correspond to a threshold cycle range from 36.5 to 15.4. Four cats tested negative by qPCR showing a discordance with the PoC test result (Lab IDs: 1128/19, 1126/20, 732/21, 868/21).

The 22 qPCR positive in U3 (LTR) cats showed at least one positive result in one of the six end-point PCR assays based on *env* gene. In particular, 19/22 cats tested positive by the end-point PCRs 1 to 4 and 15/22 tested positive by the end-point PCRs 5 and 6. Nucleotide sequences of the FeLV *env* gene amplified by end-point PCRs 1 to 4 were obtained from 15/22 qPCR-positive cats (Table 2), they were of about 1800 nucleotides in length. All these sequences showed an overall nucleotide identity of 92.1–99.6% between them and 89.9–99.9% with FeLV-A reference sequences. For 15/22 qPCR-positive cats, *env* gene nucleotide sequences were also obtained from amplicons of end-point PCRs 5 and 6 (Table 2), they were of about 1235 nucleotides in length. Ten of this 15 sequences were highly similar between them sharing a nucleotide identity of 95.5–100% with the reference sequences of the endogenous viruses. The remaining 5/15 sequences showed higher nucleotide variability (78–97%) and potentially recombination events between FeLV-A and enFeLV, compatible with FeLV-B viruses.

**Fig. 1:**
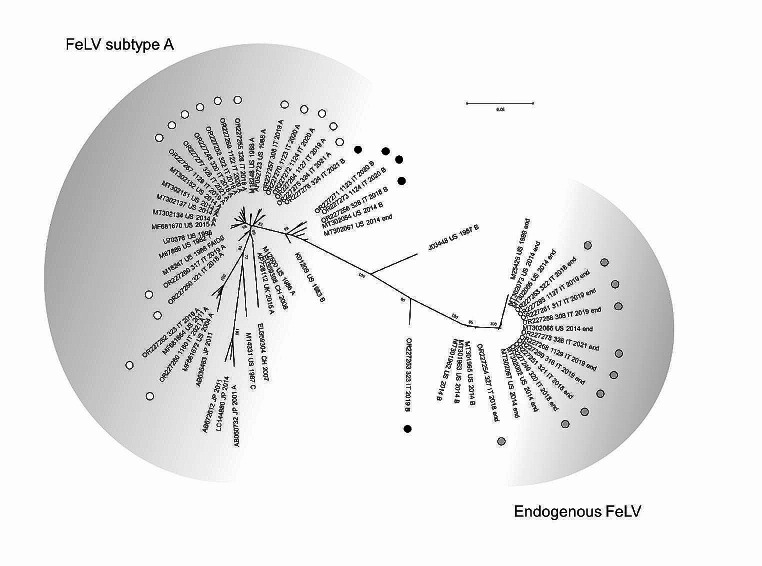
Phylogenetic tree on the nucleotide sequences of *env* gene of FeLV. Phylogeny was carried out on FeLV sequences obtained in this study and 34 reference strains (Online Resource 2) using MEGA 11 version 11.0.10. Phylogenetic tree was constructed using Neighbor-Joining method and the Tamura-Nei model with gamma distribution. Statistical support was provided by bootstrapping with 1,000 replicates and values reported on respective branch. The scale bars indicate the estimated numbers of nucleotide substitutions. Identification of the sequences undergoes the following nomenclature: GenBank accession number, strain (only for sequences obtained in this study), country (CH: Switzerland, CN: China, IT: Italy, JP: Japan, UK: United Kingdom, US: United States of America), collection date (or date of database submission), and subtype. Marked in white: sequences obtained in this study belonging to FeLV subtype A. Marked in grey: sequences obtained in this study belonging to endogenous FeLV. Marked in black: sequences obtained in this study belonging to FeLV subtype B

Phylogenetic analysis identified different clades, indicative of clusterization in FeLV subtype A, endogenous FeLV and FeLV recombinant subtype B, respectively (Fig. 1). Fifteen sequences obtained by end-point PCRs 1 to 4 clustered with the FeLV-A reference sequences from Europe, Japan and the USA. The ten highly similar sequences obtained by end-point PCRs 5 and 6 clustered with the enFeLV reference sequences. Four other sequences obtained by end-point PCRs 5 and 6 clustered with FeLV subtype B reference sequences in an intermediate position between FeLV-A and enFeLV clades while the one from cat 324/2021 grouped with but distant from other FeLV-A sequences obtained in this study.

## Discussion

In this study, 26 FeLV antigen-positive cats were tested by molecular assays to confirm the state of infection and the molecular characterization of identified FeLV was performed determining the viral subtypes circulating in Northern Italy. Signalment and history of the cats included in the study partially reflect the risk categories reported in literature: indeed only 57.7% of cats were male (Hofmann-Lehmann et al. 2018). Our data showed higher FeLV prevalence in adult cats as already reported (Levy et al. 2006; Stavisky et al. 2017), confirming that infection should be expected in adult cats as a consequence of reactivation of a previous infection and not only in kittens (Studer et al. 2019). The majority of cats included in the study had clinical signs or clinicopathological abnormalities potentially associated with FeLV infection, probably depending on the investigated cat population: sick cats visiting the VUH (Burling et al. 2017). In our study four cats resulted also positive for FIV antibodies by the PoC test: three were grouped with AC cats while one had clinical manifestations referable to retroviral infection. Considering the Italian epidemiological situation reported in the literature (Battilani et al. 2022), a lower percentage of co-infected cats was expected. On the other hand, the number of cats included in our study and the inclusion criteria adopted, do not allow to draw epidemiological conclusions.

The performance of the qPCR assay adopted in this study was not affected by the modification of the original protocol in which a probe was used instead of the SYBR green (Tandon et al. 2005), in fact FeLV provirus was detected in 84.6% of blood samples examined. Whereas, four cats tested negative, possibly as consequence of a failure of the molecular test, in case of an atypical/focal infection (Giselbrecht et al. 2023) or false positive results of the rapid PoC test adopted. These four cats had anemia or precursor-targeted immune-mediated anemia which may lead to false positive PoC antigen test results (Izquierdo Robert et al. 2023). The cause of these false positive results has not yet been definitively ascertained, although a recent study reported discordant FeLV p27 immunoassay and PCR test results in 21 cats with hematologic disorders emphasizing the importance of follow-up PCR testing in particular clinical situation like blood dyscrasia (Kornya et al. 2023). To better investigate this discrepancy, other tests could be performed, such as a second type of PoC antigen test, a laboratory-based ELISA, or a reverse-transcriptase PCR (Hofmann-Lehmann and Hartmann 2020).

The *env* gene end-point PCR assays followed by Sanger sequencing allowed to detect FeLV-A in 15 cats. In five of these cats also FeLV-B was detected, a slightly lower prevalence than reported in literature (Phipps et al. 2000). Since FeLV-B arises *de novo* after multiple recombination events of enFeLV sequences with FeLV-A, the sequence variability of this subtype depends both on recombination site involved and FeLV-A sequence diversity, thus affecting the sensitivity of diagnostic methods. This may also be one of the reasons for the low specificity of the PCRs 5 and 6 (Watanabe et al. 2013), which were expected to amplify only FeLV-B, while most of the sequenced amplicons clustered with enFeLV. Genomic variability of enFeLV coupled with its significant nucleotidic identity with exogenous FeLV makes differentiation by PCR of these two forms challenging (Chiu et al. 2018). Phylogeny of the identified FeLV-B viruses was consistent with several recombination sites at a variety of positions within the 5’ region of the *env* gene. Indeed, four of the FeLV-B viruses were intermediate between FeLV-A and enFeLV, while the one from cat 324/2021 grouped with FeLV-A, probably because the recombination break point was very close to the N-terminal of the *env* gene sequence obtained. The common but varied sites of recombination in FeLV-B variants emerged in our study support findings of other studies (Watanabe et al. 2013; Erbeck et al. 2021). This result, together with the failure to obtain *env* gene nucleotide sequences from some viruses detected by qPCR, is consistent with extensive genetic variation in the surface glycoprotein (Cano-Ortiz et al. 2022; Erbeck et al. 2021; Ortega et al. 2020; Watanabe et al. 2013).

## Conclusion

The detection of FeLV proviral DNA in 84.6% antigen-positive cats support the usefulness of qPCR assays in doubtful clinical cases. Validation of the qPCR assay on p27 antigen negative cats would be useful to evaluate its use in the diagnosis of regressive infections, particularly in blood donor cats (Nesina et al. 2015). Molecular investigation allowed to characterize 15 FeLV-A and five FeLV-B viruses, providing new information on subtypes circulating in northern Italy. With regard to the sequence variability of subtype B, it would be useful to develop a molecular tool to correctly identify this virus and proceed with further investigations to correlate this subtype with neoplasms.

**Declarations**.

### Electronic supplementary material

Below is the link to the electronic supplementary material.

Supplementary Material 1

Supplementary Material 2

## Data Availability

All data generated or analyzed during this study are included in this published article and its supplementary information files. The nucleotide sequences generated and analyzed during the current study are available in the International Nucleotide Sequence Database Collaboration repository (INSDC, http://www.insdc.org/) with the IDs: OR227248-OR227265, OR227267-OR227273, OR227275-OR227278 and OR227280.
