# Supplementary material for: Molecular investigation and genetic characterization of feline leukemia virus (FeLV) in cats referred to a veterinary teaching hospital in Northern Italy
Source: Vet Res Commun. 2024 Apr 22;48(4):2683–9. doi: 10.1007/s11259-024-10380-6 (PMC11315704; doi:10.1007/s11259-024-10380-6)
Supplement: Supplementary file 1 — Supplementary Material 1 [file 11259_2024_10380_MOESM1_ESM.docx]

***Veterinary Research Communication***

**Molecular approach to the diagnosis of Feline leukemia virus (FeLV) and genetic characterization of the viruses circulating in Italy**

Laura Gallina ^1^, Veronica Facile ^1^, Nicola Roda ^1^, Maria Chiara Sabetti ^2^, Alessia Terrusi ^1^, Lorenza Urbani ^1^, Martina Magliocca ^1^, Kateryna Vasylyeva^1^, Francesco Dondi ^1^, Andrea Balboni ^1^*, Mara Battilani ^1^

^1^ Department of Veterinary Medical Sciences, Alma Mater Studiorum-University of Bologna, Via Tolara di Sopra 40, 40064 Ozzano Emilia, Bologna, Italy.

^2^ Department of Veterinary Sciences, University of Parma, Strada del Taglio 10, 43126 Parma, Italy.

* Corresponding author:

Andrea Balboni

Department of Veterinary Medical Sciences, Alma Mater Studiorum-University of Bologna

Via Tolara di Sopra 50, 40064 Ozzano Emilia (BO), Italy

tel. +39 051 2097083

e-mail: a.balboni@unibo.it

**Online Resource 2** Reference nucleotide sequences of FeLV available in the GenBank database (https://www.ncbi.nlm.nih.gov/genbank/, accessed July 19, 2023) used for sequence analysis.

| **Strain/isolate/clone** | **Host** | **Geographic origin** | **Year ^a^** | **Nucleotide sequence** | **GenBank ID** |
| --- | --- | --- | --- | --- | --- |
| NA | cat | USA | 1987 | Subtype B | J03448 |
| GA | cat | USA | 1983 | Subtype B | K01209 |
| Glasgow-1 | cat | USA | 1986 | Subtype A | M12500 |
| Sarma | cat | USA | 1987 | Subtype C | M14331 |
| FeLV-FAIDS | cat | USA | 1988 | Subtype A (FAIDS) | M18247 |
| FeLV-3281-A | cat | USA | 1988 | Subtype A | M18248 |
| NA | cat | USA | 1989 | Endogenous | M25425 |
| NA | cat | USA | 1982 | Subtype T | M87886 |
| 81T-106 | cat | USA | 1996 | NA | U70378 |
| clone33 | cat | Japan | 2001 | NA | AB060732 |
| ON33-1 | cat | Japan | 2011 | NA | AB635483 |
| pJ7E2 | cat | Japan | 2011 | NA | AB672612 |
| Rickard subgroup A | cat | USA | 1988 | Subtype A | AF052723 |
| KI261-II | cat | Switzerland | 2007 | NA | EU359304 |
| CO265 | cat | Switzerland | 2008 | NA | EU359308 |
| Glasgow-1 | cat | UK | 2015 | Subtype A | KP728112 |
| FeLV-env431 | cat | Japan | 2014 | NA | LC144880 |
| FeLV_US_x1613_F ca2011 | cat | USA | 2011 | Subtype A | MF681664 |
| FeLV_US_x2512_Fca2015 | cat | USA | 2015 | Subtype A | MF681670 |
| FeLV_US_x1948_Pco2004 | Puma concolor | USA | 2004 | Subtype A | MF681672 |
| BDX 381_1_B | cat | USA | 2014 | Subtype B | MT301962 |
| BDX 381_7_B | cat | USA | 2014 | Subtype B | MT301963 |
| BDX 381_3_B | cat | USA | 2014 | Subtype B | MT301965 |
| BDX 399_3_B | cat | USA | 2014 | Subtype B | MT302054 |
| BDX 399_2_B | cat | USA | 2014 | Subtype B | MT302061 |
| BDX 282_1_en | cat | USA | 2014 | Endogenous | MT302062 |
| BDX 379_1_en | cat | USA | 2014 | Endogenous | MT302065 |
| BDX 381_2_en | cat | USA | 2014 | Endogenous | MT302066 |
| BDX 4457_1_en | cat | USA | 2014 | Endogenous | MT302067 |
| BDX 038_1_en | cat | USA | 2014 | Endogenous | MT302073 |
| BDX 229_2_A | cat | USA | 2014 | Subtype A | MT302134 |
| BDX 261_1_A | cat | USA | 2014 | Subtype A | MT302137 |
| BDX 400_1_A | cat | USA | 2014 | Subtype A | MT302151 |
| BDX 400_2_A | cat | USA | 2014 | Subtype A | MT302152 |

NA: not available.

a year of virus identification or sequence submission in the GenBank database.
