# Supplementary material for: Molecular investigation and genetic characterization of feline leukemia virus (FeLV) in cats referred to a veterinary teaching hospital in Northern Italy
Source: Vet Res Commun. 2024 Apr 22;48(4):2683–9. doi: 10.1007/s11259-024-10380-6 (PMC11315704; doi:10.1007/s11259-024-10380-6)
Supplement: Supplementary file 2 — Supplementary Material 2 [file 11259_2024_10380_MOESM2_ESM.docx]

***Veterinary Research Communication***

**Molecular approach to the diagnosis of Feline leukemia virus (FeLV) and genetic characterization of the viruses circulating in Italy**

Laura Gallina ^1^, Veronica Facile ^1^, Nicola Roda ^1^, Maria Chiara Sabetti ^2^, Alessia Terrusi ^1^, Lorenza Urbani ^1^, Martina Magliocca ^1^, Kateryna Vasylyeva^1^, Francesco Dondi ^1^, Andrea Balboni ^1^*, Mara Battilani ^1^

^1^ Department of Veterinary Medical Sciences, Alma Mater Studiorum-University of Bologna, Via Tolara di Sopra 40, 40064 Ozzano Emilia, Bologna, Italy.

^2^ Department of Veterinary Sciences, University of Parma, Strada del Taglio 10, 43126 Parma, Italy.

* Corresponding author:

Andrea Balboni

Department of Veterinary Medical Sciences, Alma Mater Studiorum-University of Bologna

Via Tolara di Sopra 50, 40064 Ozzano Emilia (BO), Italy

tel. +39 051 2097083

e-mail: a.balboni@unibo.it

**Online Resource 1** Primers used for molecular assays and sequencing.

| **Target and molecular method** | | **Primers** | **Nucleotide sequence (5’-3’)** | **Genome position ^a^** | **Fragment size (nucleotides) ^a^** |
| --- | --- | --- | --- | --- | --- |
| **FeLV - LTR region** | | | | | |
|  | qPCR | FeLV_U3_exo_f | AACAGCAGAAGTTTCAAGGCC |  | 131 |
|  |  | FeLV_U3_exo_r | TTATAGCAGAAAGC CGCG |  |  |
|  | End-point PCR | FeLV_standard_F | CTACCCCAAAATTTAGCGAGCTACT |  | 468 |
|  |  | FeLV_standard_R | AAGACCCCCGAACTAGGTCTTC |  |  |
| **FeLV - *env* gene** | | | | | |
|  | End-point PCR 1 | Fe-8S | CATCGAGATGGAAGGTCCAACG | 5974-5995 | 1900 |
|  |  | Fe-3R | CATGGTYGGTCYGGATCGTATTG | 7886–7908 |  |
|  | End-point PCR 2 | Fe-4S | TCCAACGCACCCAAAACCCTCT | 5989–6010 | 1900 |
|  |  | Fe-3R | CATGGTYGGTCYGGATCGTATTG | 7886–7908 |  |
|  | End-point PCR 3 | Fe-9S | GAGACCTCTAGCGGCGGCCTAC | 5711–5732 | 2400 |
|  |  | Fe-7R | GTCAACTGGGGAGCCTGGAGAC | 8174–8195 |  |
|  | End-point PCR 4 | FeLV-A Forward | ACCCAAGCTAATGCCACCTC | 6143-6162 | 1922 |
|  |  | FeLV-A Reverse | CCTCTAACTTCCTTGTATCTCATGG | 8041-8065 |  |
|  | End-point PCR 5 | PRB1 | CTGTTCACTCCTCGACAACG | Endogenous FeLV | 1400 |
|  |  | Fe-3R | CATGGTYGGTCYGGATCGTATTG | 7886–7908 |  |
|  | End-point PCR 6 | FeLV-B Forward | CAGATCAGGAACCATTCCCAGG | Endogenous FeLV | 1800 |
|  |  | FeLV-B Reverse | CCTCTATCTTCCTTGTATCTCATGG | 8041-8065 |  |
| **FeLV - *env* gene sequencing** | | | | | |
|  |  | FeLV_envseq_F | CAAGCCTCTTGGGACGGACCT | N/A |  |
|  |  | FeLV_envseq_F2 | ATGGCGGTGCTCAATTGGACC T | 7196-7216 |  |
|  |  | FeLV_envseq_R | GTGTGTACACATATTCGGGTTGAT | N/A |  |
|  |  | enFeLV_envseq_F | ACGATAAAGCTGTTCACTCCTC | N/A |  |

Primers for LTR region Real time and PCR were published by Tandon and colleagues (Tandon et al. 2005), primers for env gene exogenous FeLV PCR 1, 2, 3 and 5 were published by Watanabe and colleagues (Watanabe et al. 2013). Primers for PCR 4 and 6 were published by Erbek and colleagues (Erbeck et al. 2021). Primers used for env sequencing were designed in our laboratory.

a Primer positions and fragment size refer to the nucleotide sequence of FeLV reference strain FeLV-FAIDS (GenBank ID M18247).
